# Observational Cost-Effectiveness Analysis Using Routine Data: Admission and Discharge Care Bundles for Patients with Chronic Obstructive Pulmonary Disease

**DOI:** 10.1007/s41669-020-00207-w

**Published:** 2020-03-25

**Authors:** Padraig Dixon, William Hollingworth, Jonathan Benger, James Calvert, Melanie Chalder, Anna King, Stephanie MacNeill, Katherine Morton, Emily Sanderson, Sarah Purdy

**Affiliations:** 1 Population Health Sciences, Bristol Medical School, University of Bristol, Canynge Hall, 39 Whatley Road, Bristol, BS8 2PS UK; 2 MRC Integrative Epidemiology Unit, University of Bristol, Oakfield House, Oakfield Grove, Bristol, BS8 2BN UK; 3 Department of Health and Applied Sciences, University of the West of England, Bristol, BS16 1DD UK; 4 North Bristol Trust, Southmead Hospital, Bristol, BS10 5NB UK

## Abstract

**Background:**

Chronic obstructive pulmonary disease (COPD) is a prevalent respiratory disease, and accounts for a substantial proportion of unplanned hospital admissions. Care bundles for COPD are a set of standardised, evidence-based interventions that may improve outcomes in hospitalised COPD patients. We estimated the cost effectiveness of care bundles for acute exacerbations of COPD using routinely collected observational data.

**Methods:**

Data were collected from implementation (*n* = 7) and comparator (*n* = 7) acute hospitals located in England and Wales. We conducted a difference-in-difference cost-effectiveness analysis using a secondary care (i.e. hospital) perspective to examine the effect on National Health Service (NHS) costs and 90-day mortality of implementing care bundles compared with usual care for patients admitted to hospital with an acute exacerbation of COPD. Adjusted models included as covariates patient age, sex, deprivation, ethnicity and seasonal effects and mixed effects for site.

**Results:**

Outcomes and baseline characteristics of up to 12,532 patients were analysed using both complete case and multiply imputed models. Implementation of bundles varied. COPD care bundles were associated with slightly lower secondary care costs, but there was no evidence that they improved outcomes once adjustments were made for site and baseline covariates. Care bundles were unlikely to be cost effective for the NHS with an estimated net monetary benefit per 90-day death avoided from an adjusted multiply imputed model of −£1231 (95% confidence interval − £2428 to − £35) at a high cost-effectiveness threshold of £50,000 per 90-day death avoided.

**Conclusion and Recommendations:**

Care bundles for COPD did not appear to be cost effective, although this finding may have been influenced by unmeasured variations in bundle implementation and other potential confounding factors.

**Electronic supplementary material:**

The online version of this article (10.1007/s41669-020-00207-w) contains supplementary material, which is available to authorized users.

## Key Points for Decision Makers

**Table:** 

Care bundles for chronic obstructive pulmonary disease (COPD) are a set of standardised, evidence-based interventions that may improve outcomes in hospitalised COPD patients.
We estimated the cost effectiveness of care bundles for acute exacerbations of COPD using routinely collected observational data. We compared hospital costs with 90-day mortality in 14 hospitals that implemented care bundle interventions to varying degrees.
COPD care bundles were associated with slightly lower secondary care costs, but there was no evidence that they improved mortality. There was no evidence that care bundles were likely to be cost effective for COPD.

## Introduction

Chronic obstructive pulmonary disease (COPD) refers to set of long-term respiratory diseases that encompass several pathologies that affect the lungs and inhibit airflow, including chronic bronchitis and emphysema. The prevalence of COPD in the United Kingdom (UK) is estimated to constitute 1.2 million diagnosed cases [1], and up to 2 million undiagnosed cases [2]. The condition is associated with approximately 30,000 deaths annually [1] in the UK.

Managing COPD in secondary care requires substantial resources. In the UK, COPD is responsible for 12% of all ambulatory care sensitive hospital admissions [3]. Some 23% of patients admitted for COPD will be re-admitted for COPD at least once within 90 days of discharge [4], and the condition is the single most common cause of readmission in the NHS [5]. The financial sustainability of publicly funded healthcare services, such as the National Health Service (NHS) in the UK, depends in large part on the efficient management of chronic conditions—such as COPD—that are associated with substantial numbers of potentially avoidable admissions and readmissions.

Care bundles may improve patient outcomes and reduce the resources required to manage COPD. These bundles are sets of simple, structured, evidence-based clinical actions or interventions intended to improve patient outcomes [6] and are administered as part of inpatient hospital care. Admission care bundles and discharge care bundles for COPD were developed by the British Thoracic Society in conjunction with NHS Improvement [7].

The admission bundle, administered at hospital admission, is intended to facilitate coordinated care for admissions following an acute exacerbation of COPD. For example, an important first step in this bundle is correctly establishing a diagnosis of an acute exacerbation of COPD, supported by electrocardiogram and X-ray. The discharge bundle is primarily intended to reduce re-admissions by ensuring appropriate assessment prior to discharge and to ensure that patients are confident in their use of medications. For example, patients should have respiratory medicines reviewed and inhaler technique assessed before leaving hospital [8].

Specimen examples of admission and discharge bundles are available here (admissions: https://www.brit-thoracic.org.uk/document-library/quality-improvement/copd/copd-admission-care-bundle/discharge: https://www.brit-thoracic.org.uk/document-library/quality-improvement/copd/copd-discharge-care-bundle/) from the British Thoracic Society. The elements of each of these bundles are as follows:

Admission bundle:

- Ensure correct diagnosis of an acute exacerbation of COPD
- Assess oxygen and prescribe target range
- Recognise and respond to respiratory acidosis
- Administer steroids and nebulisers within 4 h of admission
- Respiratory team review within 24 h

Discharge bundle:

- Review medication and demonstrate inhaler use
- Provide self-management plan and emergency drug pack
- Assess and offer referral for smoking cessation
- Assess suitability for pulmonary rehabilitation
- Arrange follow-up call within 72 h of discharge

Note that these are examples of what might constitute the elements of admission and discharge bundles, and local variations are possible and were observed during the study. However, in all cases, the individual elements of each care bundle are generally based on clinical evidence, but there is little to no evidence as to whether the use of COPD care bundles themselves in routine clinical settings might be cost effective. We therefore estimated the cost effectiveness of both admission and discharge care bundles using observational patient-level data from 14 hospital sites located in England and Wales. This cost-effectiveness study was part of a wider project evaluating the effectiveness of care bundles for acute exacerbations of COPD [8–10].

## Methods

Acute hospitals with an emergency department and providing adult respiratory in-patient care in England and Wales formed the target population for the study. Following expressions of interest in participating in this study, 14 hospitals were allocated to either an ‘implementation’ group or a ‘comparator’ group. Hospitals in the implementation group delivered both admission and discharge bundles. Comparator hospitals were selected for the implementation hospitals based on a number of pre-specified criteria including the number of COPD admissions, 28-day re-admission rates and COPD mortality rates. This resulted in seven implementation and seven comparator sites. Data were analysed using a controlled before-and-after study design [8].

### Data Collection

Data from all sites were requested. We aimed to collect 24 months of data, encompassing the 12 months immediately preceding index date (the date of the introduction of COPD care bundles in the implementation group and the equivalent date in the comparator) and the 12-month period following their introduction. Index dates for the introduction of both admission and discharge bundles at implementation sites ranged between 2013 and 2015. Comparator sites were allocated index dates on a comparable basis to those of the implementation sites.

Sites provided pseudo-anonymised patient-level data including age, sex, ethnicity, ICD-10 diagnosis codes [11], admission data and 90-day mortality via linkage to death registry information for all patients admitted for an acute exacerbation of COPD (ICD-10 diagnostic codes J41–J44) during the study period. The use of elements of COPD care—such as non-invasive ventilation—was requested from the medical records of a random sample at each site of 140 adult patients admitted for an acute exacerbation of COPD.

### Cost Perspective for Economic Analysis

We adopted a secondary (hospital) health system (i.e. NHS) perspective for costs, which were expressed in 2015/16 prices and were not discounted over the 12 months before and after the index date.

### Measurement and Valuation of Resource Use

Sites provided patient-level data for individuals admitted to hospital (‘admitted patient care’) with a primary diagnosis of COPD during the study period. In addition to admitted patient care, we also collected data on critical care, emergency care and outpatient care. We assumed that there was no systematic difference between patients attending comparator and implementation hospitals in the use of COPD-related hospital care outside those included in our analysis.

Resource use was costed for each care type using the Healthcare Resource Groups (HRGs) produced by application of Reference Cost Grouper software [12] for each financial year.^1^ See Appendix 2 in the electronic supplementary material for further detail on the data processing steps involved. HRGs reflect groups of similar activity undertaken (based on OPCS-4 [13] procedure codes) for similar diagnoses (based on ICD-10 diagnosis codes).

The Grouper software was used to convert information concerning patient procedures, diagnoses and other data into ‘Finished Consultant Episode’ HRGs. HRGs were linked to unit costs reported in NHS Reference Costs [14]. Costs relating to critical care, emergency care and outpatient care were included only if they occurred on or after the date of the first included inpatient COPD-related admission. Diagnostic information pertaining to outpatient care is relatively limited, and for many patients, not all outpatient attendances after the index inpatient admission will relate to COPD. We therefore undertook a sensitivity analysis in which we calculated hospital costs excluding the costs of outpatient attendance. Costs for longer-staying patients were reported as the sum of the Finished Consultant Episode cost and the sum of per diem excess bed day costs.

Data collected from the medical records on patient use of discrete elements of COPD care were used to compare the costs of these elements between implementation and comparator sites on an ‘available case’ or ‘complete case’ basis. This analysis was conducted separately from the main cost-effectiveness analysis because the costs of many individual elements are likely included in the HRG costing analysis. Element-specific resource use was valued using NHS Reference Costs [14], the NHS drug tariff [15], Unit Costs of Health and Social Care [16] and/or from published literature. Further details are provided in Appendix 3 of the electronic supplementary material.

### Outcome Measurement

The proportion of patients alive at 90 days following the index admission was used as the outcome for effectiveness in the economic analysis. The cost-effectiveness results may therefore be interpreted as the incremental cost per percent change in the proportion of patients surviving until at least day 90.

### Accounting for the Observational Study Design

Allocation of sites to implement care bundles was not random and estimates of cost effectiveness of COPD bundles were at risk of bias because patient costs and survival may systematically differ between implementation and comparator sites and over time for reasons unconnected to the administration of a care bundle. Moreover, there was some degree of prior implementation of care bundles in some of the comparator sites, although we did not have access to the precise timing and nature of this implementation. The attempt to have comparable sites in both groups provides a partial but incomplete means of reducing this bias.

To mitigate other risks of bias due to confounding, we attempted to follow wherever possible the checklist criteria of Kreif et al. [17] for observational cost-effectiveness analyses. A summary version of the checklist and the methods used to comply with its recommendations is available in Appendix 1 of the electronic supplementary material. We examined whether there were baseline covariates that predicted the use of care bundles by plotting histograms for age at admission and quintiles of deprivation, and by calculating standardised differences [18] for sex and ethnicity.

The regression models estimated implicitly embody a ‘parallel trends’ assumption in their analysis of the before and after data: that pre-bundle trends in costs and mortality were similar between each site type, and that comparator sites were not affected by the introduction of bundles at implementation sites. Using the preferred final specification on complete case data, we also estimated a ‘placebo regression’ to test this assumption. We assumed care bundles were introduced at implementation sites half-way through the year before their actual introduction. If the parallel trend assumptions hold, then no significant effect on net benefit and on the interaction between time period and site type should be evident before care bundles were introduced, in the absence of chance and a systematic difference between the implementation and comparator sites.

### Regression Analysis

Two types of regression model were estimated to account for the sensitivity of the cost-effectiveness conclusions to structural uncertainty associated with the choice of statistical method: seemingly unrelated regressions (SURs), and net benefit regression. We estimated univariate net benefit regression models [19], in which patient-level incremental net benefit was regressed on a treatment indicator and covariates according to specification. We estimated SURs in which cost and mortality data were separately modelled but with a correlated error structure. We also explored generalised linear models, but these models did not converge except for the simplest specifications and are not discussed further.

All regression models included an interaction between study period (before and after the introduction of care bundles) and site type (implementation and comparator sites), in which the treatment effect of care bundles is the coefficient on the interaction between study period and site type. We attempted to estimate each type of regression under three specifications: unadjusted for any covariates other than the period/site interaction, adjusted for the month when bundles were introduced and a mixed effect for each hospital trust, and finally a fully adjusted model accounting for month of bundle introduction, mixed effect for hospital trust, and baseline covariates. These baseline covariates comprised age at admission, sex, ethnicity (coded as ‘white’ and ‘other’ due to low frequencies in non-white categories), and deprivation quintile based on the Index of Multiple Deprivation associated with the postcode of the patient’s residence. Mixed effect terms are random effects intended to capture variation not explained by these other covariates. These models therefore comprise a mix between the ‘fixed’ effects such as age and sex and the random effects that reflect other sources of variation.

### Reporting of Results

Cost-effectiveness results were expressed using net monetary benefit (NMB) statistics, calculated as:

$${\text{NMB}} = \Delta {\text{survival}}*\lambda - \Delta {\text{costs}} .$$

The $\Delta$ term is the incremental difference operator; $\Delta {\text{costs}}$, for example, represents the cost difference between implementer and comparator sites. The lambda ($\lambda )$ term represents the cost-effectiveness threshold. This captures the rate at which the health system converts monetary resources into avoided 90-day mortality. Different threshold values (£5000, £10,000, £20,000, £30,000 and £50,000) were used in estimating NMB in the absence of a specific 90-day survival cost-effectiveness threshold in the NHS. This information can be used by decision makers to assess whether the incremental secondary care costs of care bundles and associated changes in the proportion of patients alive at 90 days might constitute a cost-effective use of health system resources.

Net benefit statistics, confidence intervals (CI) on net benefit statistics and cost-effectiveness acceptability curves (CEACs) were calculated parametrically from the SUR and net benefit regressions.

### Missing Data

We implemented multiple imputation by chained equations in Stata 14 using the—ice—command [20, 21] to account for missing data, including for cost data that could not be assigned to an HRG code. The imputation model was stratified by site type and included all baseline variables, cost data, site, site type, and the survival outcome. Predictive mean matching [20] was used to account for non-Gaussian distributions. The number of imputed data sets (*n* = 40) created was chosen to be at least 100 times greater than the proportion of missing data [20]. The methods of Faria et al. [22] were used to reflect variation within and between the imputed datasets use in regression analysis. All analyses were conducted in Stata v14 (Statacorp: College Station, TX, USA).

### Qualitative Analysis

The quantitative cost-effectiveness analysis was complemented by two additional pieces of information obtained from qualitative interviews and observations from a subset of study sites. First, the duration of interactions between clinical staff (doctors, nurses, health care assistants, and others) and COPD patients was observed on medical admissions units, acute wards and/or general wards. Patients were observed for up to 2 h to record the duration of any interactions between the patient and hospital staff. Second, a small sample of patients, selected purposively, were interviewed concerning their post-discharge engagements with different healthcare resources. Further details are reported in Appendix 6 of the electronic supplementary material.

## Results

Data from 12,532 patients undergoing emergency hospital admissions during the study period were analysed in the economic evaluation. The 14 sites providing these data were located across England and Wales and included district hospitals and larger city hospitals.

### Covariate Balance and Overlap

The results of covariate overlap and balance tests, described in more detail in Appendix 1 of the electronic supplementary material, were considered to be acceptable.

### Missing Data

A total of 14 sites were recruited to provide individual level data. One site changed record systems 6 months into the study period and only provided 4 months of pre-index date data. Four sites provided data missing the last 1–2 months of 90-day follow up. Two sites did not provide 90-day mortality outcomes for the last 90 days of follow-up. Regression models were estimated including a ‘month in year’ effect to account for the imbalances to which this gave rise.

Data on patient age and sex were complete. Ethnicity was missing for 2.9% of participants included in the analysis sample, and deprivation data were missing for 1.6% of participants.

Nine of the 14 sites provided incomplete case note extraction data. Two sites from the same NHS Trust did not have capacity to complete two full sets of the case note extraction audit forms (i.e. 140 sets of notes each). As a compromise, they performed the case note extraction on 70 sets of notes at each of the two sites. This reduced the total number of case report forms (CRFs) that we received; seven other sites also failed to provide the full 140 case note extraction CRFs due to limited time and resources.

There were instances of missing cost data within each type of hospital care (Table 1). A total cost variable was created per individual by summing across these four cost categories, of which some 31.8% of data (34.2% comparator sites, 29.3% implementation sites) were coded as missing. Total cost data were more likely to be missing for comparator sites than implementation sites (odds ratio 0.80, 95% CI 0.74–0.86).

**Table 1:** Missing cost data

	All sites % missing (*n*)	Comparator sites % missing (*n*)	Implementation sites % missing (*n*)
Admitted patient care	8.29 (1039)	2.07 (130)	14.53 (909)
Critical care	21.99 (2754)	30.78 (1932)	13.14 (822)
Emergency department	2.21 (277)	1.23 (77)	3.20 (200)
Outpatient	6.76 (847)	0.11 (7)	13.43 (840)

### Cost and Mortality Data

Complete total cost data is summarised in Table 2, and for multiply imputed data in Table 3.

**Table 2:** Summary of complete cost data per patient

	Comparator sites	Implementation sites	Difference (95% CI)^a^
	Mean cost (SD)	Mean cost (SD)	
Total cost per patient	£5454 (5058) *N* = 4130	£5769 (8425) *N* = 4423	£315 (18 to 612)
Total cost per patient in pre-bundle period	£5987 (5974) *N* = 2338	£6692 (8574) *N* = 2372	£705 (283 to 1128)
Total cost per patient in post-bundle period	£4759 (3399) *N* = 1792	£4702 (8122) *N* = 2051	−£57 (− 461 to 347)

*CI* confidence interval, *SD* standard deviation

^a^Confidence intervals calculated from unadjusted linear regression

**Table 3:** Summary of imputed cost per patient

	Comparator sites mean cost (SE)^a^ *N* = 6276	Implementation sites mean cost (SE) *N* = 6256	Difference (95% CI)^b^
Total cost per patient	£6750 (130)	£5356 (131)	−£1395 (−1757 to −1034)
Total cost per patient in pre-bundle period	£7398 (189)	£6070 (185)	−£1328 (−1848 to −809)
Total cost per patient in post-bundle period	£6057 (172)	£4472 (183)	−£1584 (−2072 to −1097)

*CI* confidence interval, *SE* standard error

^a^Standard errors rather than standard deviations are reported for multiply imputed data

^b^Confidence intervals calculated from unadjusted linear regression

An important difference between the imputed and completed case data sources is that all 14 sites are represented in the imputed data. This is likely to be the biggest driver of differences in point estimates, which indicate higher mean levels of cost and relatively high differences between site types in the imputed compared with the complete case data. The proportion of patients alive at 90 days (Table 4) was slightly higher at implementation sites both before and after the introduction of care bundles. Given negligible amounts of missing day in 90-day survival, imputed point estimates (and therefore point estimates of difference between site type) of these proportions were the same to two decimal places as the available case data in Table 4.

**Table 4:** Proportion of patients alive at 90 days (complete cases)

	Comparator sites mean proportion (SD)	Implementation sites mean proportion (SD)	Difference (95% CI)^a^
Proportion	0.90 (0.29) *N* = 6276	0.92 (0.27) *N* = 6256	0.02 (0.01–0.03)
Proportion in pre-bundle period	0.90 (0.30) *N* = 3245	0.92 (0.38) *N* = 3458	0.02 (0.00–0.03)
Proportion in post-bundle period	0.91 (0.29) *N* = 3031	0.93 (0.26) *N* = 2798	0.02 (0.01–0.03)

*CI* confidence interval, *SD* standard deviation

^a^Confidence intervals calculated from unadjusted linear regression

### Performance of Different Estimators and Model Selection

Estimation difficulties due to model convergence issues and data sparsity limited model selection. Nevertheless, we explored Akaike Information Criterion (AIC) and Bayesian Information Criterion (BIC) statistics for SUR and net benefit models estimated on complete cases. We also present residual plots from complete case adjusted net benefit regression model in Appendix 5 of the electronic supplementary material. Net benefit and SUR models produced broadly similar results; results from net benefit regressions are reported below and results from SUR models in the electronic supplementary material (see Appendix 4).

### Results of Cost-Effectiveness Analysis

The probability of cost effectiveness attenuated on inclusion of covariates (Table 5). These results are similar to those of the SUR models reported in the electronic supplementary material. Both the AIC and the BIC were slightly lower under the fully adjusted net benefit models (AIC 170,741; BIC 170,909) than the fully adjusted SUR models (AIC 169,899; BIC 170,261).

**Table 5:** Net monetary benefit at alternative values of the cost-effectiveness threshold—complete case analysis using net benefit regression

Models estimated	Net benefit regression, unadjusted *N* = 8553			Net benefit regression, adjusted for month in year and mixed effect for trust cluster *N* = 8553			Net benefit regression, adjusted for month in year, mixed effect for trust cluster, and all baseline covariates (age, sex, ethnicity and deprivation) *N* = 8121		
	Comparator mean	Implementation mean	Interaction (95% CI)^a^	Comparator mean	Implementation mean	Interaction (95% CI)^b^	Comparator mean	Implementation mean	Interaction (95% CI)^a^
Net monetary benefit at *λ* = £20,000^b^									
Monetary benefit in ‘pre’ period	£11,926	£11,242	£884 (117 to 1650)	£11,826	£11,361	£764 (2 to 1527)	£11,846	£11,336	£798 (15 to 1581)
Monetary benefit ‘post’ period	£13,411	£13,611		£13,400	£13,599		£13,396	£13,684	
Cost-effectiveness statistics^c^									
NMB at *λ* = £5000 (95% CI)			£792 (190 to 1395)			£699 (99 to 1300)			£784 (167 to 1402)
Probability cost effective at *λ* = £5000			1.00			0.99			0.99
NMB at *λ* = £10,000 (95% CI)			£823 (186 to 1460)			£721 (87 to 1355)			£789 (137 to 1441)
Probability cost effective at *λ* = £10,000			0.99			0.99			0.99
NMB at *λ* = £30,000 (95% CI)			£945 (− 1 to 1891)			£809 (− 133 to 1751)			£808 (− 158 to 1774)
Probability cost effective at *λ* = £30,000			0.97			0.95			0.95
NMB at *λ* = £50,000 (95% CI)			£1,067 (− 309 to 2442)			£899 (− 473 to 2271)			£829 (− 576 to 2234)
Probability cost effective at *λ* = £50,000			0.94			0.90			0.88

*CI* confidence interval, *ICER* incremental cost-effectiveness ratio, *NMB* net monetary benefit, *λ* cost-effectiveness threshold value

^a^Note that for net benefit regression, the interaction measures net benefit, and is reported above for a threshold value of £20,000

^b^Threshold values represent cost per death avoided at 90 days

^c^Interactions measure the difference in outcomes (either cost or 90-day survival) between (outcomes at implementation sites minus outcomes at comparator sites in the ‘pre’ period) and (outcomes at implementation sites minus outcomes at comparator sites in the ‘after’ period)

**Fig. 1:**
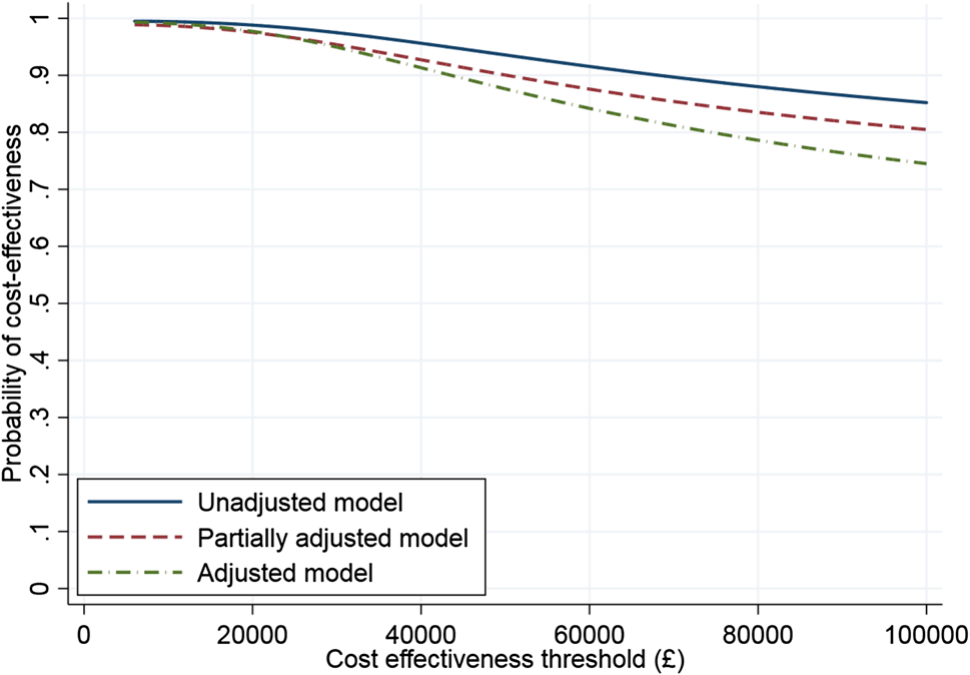
Cost-effectiveness acceptability curves (CEACs) for complete case net benefit models

We also ran two placebo regressions that estimated SUR and net benefit models on fully adjusted complete case data. Interaction effects in each model between site type and time period included the null. Moreover, estimated ‘cost effectiveness’ measured using net benefit was much smaller than in the base case (net benefit model at a threshold of £20,000 = £233 [95% CI − 845 to 1311] compared with £798 in the base case). Figure 1 summarises the associated CEACs of the complete case analysis.

Complete case estimates may be biased (because of the exclusion of entire sites but also the exclusion of individual patient records in some cases) and inefficient (by excluding responses from over 31% of all individuals included in the sample). Table 6 presents results from the unadjusted, partially adjusted and fully adjusted models estimated with net benefit regression on imputed data.

**Table 6:** Net monetary benefit at alternative values of the cost-effectiveness threshold–imputed cases using net benefit regression

Models estimated	Net benefit regression, unadjusted model *N* = 12,532 imputed observations			Net benefit regression, adjusted for month in year, and trust site as a mixed effect *N* = 12,532 imputed observations			Net benefit regression, adjusted for month in year, trust site as a covariate, and baseline variables *N* = 12,532 imputed observations		
	Comparator mean	Implementation mean	Interaction (95% CI)	Comparator mean	Implementation mean	Interaction (95% CI)	Comparator mean	Implementation mean	Interaction (95% CI)^a^
Monetary benefit and net monetary benefit at *λ* = £20,000									
Monetary benefit in ‘pre’ period	£10,580	£12,270	£282 (− 524 to 1088)	£9942	£12,380	−£1099 (− 1902 to − 296)	£9997	£12,356	−£1,089 (− 1891 to − 297)
Monetary benefit in ‘post’ period	£12,148	£14,119		£12,867	£14,206		£12,898	£14,168	
Cost-effectiveness statistics^a^									
NMB at *λ* = £5000 (95% CI)			£263 (− 452 to 977)			−£1019 (− 1773 to − 305)			−£1013 (− 1727 to − 299)
Probability cost effective at *λ* = £5000			0.76			0.00			0.00
NMB at *λ* = £10,000 (95% CI)			£269 (− 464 to 1002)			−£1046 (− 1778 to − 315)			−£1039 (− 1770 to − 308)
Probability cost effective at *λ* = £10,000			0.75			0.00			0.00
NMB at *λ* = £30,000 (95% CI)			£294 (− 622 to 1210)			−£1150 (− 2063 to − 237)			−£1137 (− 2048 to − 227)
Probability cost effective at *λ* = £30,000			0.74			0.01			0.01
NMB at *λ* = £50,000 (95% CI)			£319 (− 884 to 1523)			−£1249 (− 2452 to − 45)			−£1231 (− 2428 to − 35)
Probability cost effective at *λ* = £50,000			0.70			0.02			0.02

a Threshold values represent cost per death avoided at 90 days

*CI* confidence interval, *NMB* net monetary benefit, *λ* = cost-effectiveness threshold value

Imputed regressions (Table 6) exhibit a declining probability of cost effectiveness, with the degree of attenuation on adjustment much more pronounced than in the complete case analysis. The partially and fully adjusted imputed models result in CEACs that were practically indistinguishable from the horizontal axis and hence are not displayed.

Overall, results were sensitive to imputation and adjustment for covariates. Assuming that the imputation model is less biased than complete case models, this suggests that, once all sites are included in the analysis and adjustment is made for available covariates, there is little probability of care bundles being cost effective at any cost-effectiveness threshold. A sensitivity analysis excluding outpatient costs from the fully adjusted, imputed analysis was undertaken. This slightly reduced the estimated probability of care bundles being cost effective but overall results were similar to the base-case analysis.

We also conducted a simple threshold analysis to identify whether there was any level of costs at which net benefit would likely be positive for the fully adjusted imputed analysis. For a cost-effectiveness threshold of £20,000 per 90-day death averted, a 90% reduction in cost increased the probability of care bundles being cost effective to 17%, and a 99% reduction increased the probability to 29%. This makes clear that, given the mortality outcomes observed, there is no plausible reduction in costs that would make the intervention more likely to be cost effective than not.

### Elements of Care Bundles

A comparison between site types in the costs of care bundle elements is reported in Table 7. The sum of costs reported is on an element-by-element complete case base. Appendix 3 in the electronic supplementary material provides further detail on the costs of different elements and the sources used to construct them.

**Table 7:** Costs of elements of care bundles

	Mean cost (SD)
All bundle elements	
Comparator sites	£298.32 (175.95)
Implementation sites	£349.91 (166.34)
All sites	£325.45 (172.89)
Admission bundle elements only	
Comparator sites	£279.22 (168.27)
Implementation sites	£312.90 (160.10)
All sites	£296.78 (164.87)
Discharge bundle elements only	
Comparator sites	£19.60 (24.71)
Implementation sites	£37.01 (33.28)
All sites	£28.68 (30.74)

*SD* standard deviation

More costs are attributable to the admission care bundle than to the discharge bundle. It is notable that comparator sites incurred many of the costs associated with providing elements of care bundles, although these costs are lower than at implementation sites. This is consistent with evidence [9] that comparator sites also engaged in many of the ‘bundle’ activities undertaken by implementation sites.

## Discussion

### Strengths and Limitations

To our knowledge, this is the first study to estimate the cost effectiveness of care bundles for acute exacerbations of COPD. A large sample of detailed patient-level data was analysed under an observational design, complemented by comparisons of the use and cost of different elements of care bundles, and by qualitative assessments (see Appendix 6 of the electronic supplementary material) of the volume of care before and after discharge. This amounts to a diverse range of sources and methodological perspectives and reflects an effort to engage with ‘real-world evidence’ in the form of existing data sources and contemporary clinical practice.

The study has a number of limitations, many of which stem from the use of data sources not collected for research purposes. A fundamental and unavoidable challenge was that care bundles were implemented to varying degrees, at varying times by hospitals with different local cultures, management practices and patient compositions. The reliability of care bundle implementation was not fully observable to the research team, particularly given the retrospective design used, and the absence of evidence in favour of cost effectiveness may reflect a lack of efficacy or a lack of implementation to a degree at which efficacy could be reliably judged in comparative inferential analysis.

This limitation also complicates the interpretation of the cost-effectiveness results. The comparison in this analysis is not between ‘care bundles implemented’ and ‘care bundles not implemented’. Instead, claims concerning the cost effectiveness of care bundles should be interpreted as a comparison between greater (at implementation sites) and lesser (at comparator sites) degrees of implementation. The results do not identify the cost effectiveness of care bundles per se, but rather the cost effectiveness of a more rather than less comprehensive implementation.

A limited number of baseline covariates were available, and confounding of the relationship between care bundles and the outcomes studied cannot be discounted, despite efforts to adhere to best practice in observational cost-effectiveness analysis. For example, we did not have access to individual-level data on smoking history, which is the most important risk factor for incident COPD. Moreover, despite our expectation that some missing data would be encountered given the observational research design, data availability was less than expected, despite efforts from the research team and staff at participating sites to secure access to data.

Comparisons between the complete case and imputed analysis were therefore complicated by the absence of data from entire sites for the former analysis models. However, given the comprehensive availability of mortality data, and that little difference was observed between types of site in relation to mortality, the precise details of the imputation are unlikely to have affected our overall conclusions regarding the outcome.

The reliance on 90-day mortality as the measure of effectiveness of this analysis cannot identify other outcomes that may be relevant to the cost effectiveness of care bundles. Quality-of-life data is not routinely collected in the clinical environments from which we drew our data. This has the important consequence that we could not calculated quality-adjusted life-years (QALYs), which are the preferred outcome measure for cost-effectiveness analysis [23] performed in the jurisdictions we study. It is possible that data on QALYs might not have changed our overall conclusions, given the modest mortality difference observed, and given the finding from the systematic review of van der Schans et al. [24] that the cost effectiveness of interventions for COPD tends to be driven by exacerbations and mortality. Nevertheless, the lack of quality-of-life data is an important limitation on our analysis.

The ward observations and post-discharge interviews concerning community resource use, described in Appendix 6 of the electronic supplementary material, offered limited evidence concerning the impact of care bundles on healthcare costs. Some planned models could not be estimated, either because of sparsity in models with many indicator variables, or because of convergence issues when estimating mixed effects models. Placebo models testing the sensitivity of results to the timing of care bundle introduction were null. This offers a degree of reassurance that an ‘effect’ of care bundles on cost effectiveness is not obvious if they are modelled as having been introduced 6 months before their actual introduction and is some evidence that the parallel trends assumption may be reasonable. However, the nature of placebo tests means that these results are necessarily suggestive rather than definitive since the absence of an ‘effect’ in the placebo test does not mean that the primary estimation models are themselves necessarily well posed, nor that the absence of evidence is evidence of absence.

## Conclusions

The economic analysis of patient records from up to 12,532 individuals receiving care indicated that a wider use of COPD care bundles was associated with slightly lower secondary care costs, but there was no evidence that this improved outcomes. Interpretation of these quantitative results is complicated by incomplete control of potential confounding variables, and partial implementation of care bundles. Patient observation and patient interviews with a small sample of individuals did not reveal any gross differences in resource use between site types.

Overall, observational analysis of these various sources of evidence using different methodological tools did not identify strong evidence that an extensive implementation of care bundles is likely to be cost effective when compared with less extensive implementation for the NHS in this patient group.

## Electronic Supplementary Material

Below is the link to the electronic supplementary material.

Supplementary material 1 (DOCX 164 kb)

## Data Availability

Consent for patient-level data sharing was not obtained. Requests for anonymised summary data should be made to Professor Sarah Purdy.

## Footnotes

1 Some sites reported data in the 2016/17 financial year, which was valued in 2015/16 terms using the Grouper and Reference Cost data for that year.
