# Supplementary material for: Observational Cost-Effectiveness Analysis Using Routine Data: Admission and Discharge Care Bundles for Patients with Chronic Obstructive Pulmonary Disease
Source: Pharmacoecon Open. 2020 Mar 25;4(4):657–67. doi: 10.1007/s41669-020-00207-w (PMC7688870; doi:10.1007/s41669-020-00207-w)
Supplement: Supplementary file 1 — Supplementary material 1 (DOCX 164 kb) [file 41669_2020_207_MOESM1_ESM.docx]

Observational cost-effectiveness analysis using routine data: Admission and discharge care bundles for patients with chronic obstructive pulmonary disease

***PharmacoEconomics - Open***

Padraig Dixon*^1,2^, William Hollingworth^1^, Jonathan Benger^3^, James Calvert^4^, Melanie Chalder^1^, Anna King^1^, Stephanie MacNeill^1^, Katherine Morton^3^, Emily Sanderson^1^ Sarah Purdy^1^

* Corresponding author

Padraig.dixon@bristol.ac.uk

1 Population Health Sciences, Bristol Medical School, University of Bristol, Canynge Hall, 39 Whatley Road, Bristol, BS8 2PS

2 MRC Integrative Epidemiology Unit, University of Bristol, Oakfield House, Oakfield Grove, BS8 2BN

3 Department of Health and Applied Sciences, University of the West of England, Bristol. BS16 1DD

4 North Bristol Trust, Southmead Hospital, BS10 5NB

**Running title**: Cost-effectiveness of COPD care bundles

This supplementary appendix is organised as follows.

- Appendix 1 presents the Krief et al checklist for observational cost-effectiveness analysis and describes the covariate and overlap balance
- Appendix 2 describes data processing steps on Hospital Episode Statistics data prior to the application of the Grouper software
- Appendix 3 describes the costing of elements of care bundles
- Appendix 4 presents results from Seemingly Unrelated Regression (SUR) models
- Appendix 5 presents model fit graphs from available case net benefit models
- Appendix 6 describes the methods and results of the qualitative analysis
- Appendix 7 contains a completed CHEERS checklist

Appendix 1 – Krief et al checklist and covariate overlap and balance

| Question | Explanation of question | Examples of recommended methods | Methods applied |
| --- | --- | --- | --- |
| Question 1a – Did the study assess the ‘no unobserved confounding’ assumption? | In the difference-in-difference methodological context of this study, this assumption implies that there are no time-varying confounders correlated with treatment assignment and study endpoints. | (Full assessment) Causal diagrams or mathematical description of the relationships by structural equation models, use of placebo tests  (Partial assessment) Substantive *a priori* knowledge, commentary on plausibility and sufficiency of observed confounders | Placebo tests and qualitative commentary. |
| Question 2 – Did the study assess whether the baseline covariates (e.g. age and sex) had distributions that overlapped between the treatment groups? | Good overlap suggests that there are no baseline covariates that fully predict allocation to care bundles or no care bundles | (Full assessment) Histograms or smoothed density plots of the continuous covariates, standardised differences investigated for binary variables  (Partial assessment) Inspecting standardised differences of variables to assess overlap | Summary statistics of baseline covariates by site type. Histograms and kernel densities calculated for continuous covariate (age), standardised differences calculated for binary covariates. |
| Question 3 – Did the study assess the specification of the regression model for (i) health outcomes and (ii) cost? | Parametric regression is unbiased and efficient only if correctly specified. | (Full assessment): Statistical tests to assesses specification of regression models, such as link tests for generalised linear models (GLM), penalised likelihood statistics, residual plots for OLS  (Partial assessment) Qualitative arguments, e.g. consensus in literature, linear net benefit modelling, distribution of an outcome implies a specific modelling approach etc. | Consideration of penalised likelihood statistics, and qualitative arguments. |
| Question 5 – Did the study consider structural uncertainty arising from the choice or specification of the  statistical method for addressing selection bias? | This criterion is fully met if the authors conducted an additional statistical analysis beyond the primary method used to address selection bias and interpreted how the results are altered by using the alternative  method. | (Examples of full assessment) Different specifications applied to cost and effectiveness data, e.g. OLS versus Gamma GLM  (Partial assessment) Statistical analysis beyond the primary method, omit/include some variables from regression models as a sensitivity analysis, discussion of suspected bias. | Additional statistical analyses conducted beyond the primary method.  Separate models applied to cost and effectiveness data for some specifications  Assessment of effect of including different covariates |

Table A1: Checklist for observational cost-effectiveness analysis

Notes: Question 1(b) in the checklist refers to instrumental variable analysis, and is not relevant to the present study. Likewise, Question 4 in the checklist relates to regression analysis following matching – matching was intended to be used in this study, but was not deployed for the reasons described.

Covariate overlap and balance tests were undertaken. These comparisons indicate that the means, ranges and distributions of age at admission are broadly similar at each site type. The binary sex and ethnicity variables also showed reasonable balance (Table A2), albeit exhibiting a slight difference between site types in ethnicity (standardised difference >0.10)

Table A2: Covariate balance for binary baseline covariates

|  | **Comparator sites** | | **Implementation sites** | | **Balance** | |
| --- | --- | --- | --- | --- | --- | --- |
|  | **Mean** | **Variance** | **Mean** | **Variance** | **Standardised difference** | **Variance ratio** |
| sex^1^ | 1.51 | 0.25 | 1.51 | 0.25 | 0.00 | 1.00 |
| ethnicity^2^ | 1.07 | 0.06 | 1.10 | 0.09 | 0.11 | 1.4 |

Notes (1) Sex is coded as 1=Males, 2=Female. (2) Ethnicity is coded as 1=White, 2=Other.

Histograms were calculated for quintiles of deprivation (Figure A2), and a histogram with kernel density was calculated for age (Figures A3 and A4) as the only continuous baseline covariate.

Figure A3: Deprivation by site type

Figure A4: Comparison of age at admission by site type

Figure A5: Kernel densities of age by site type as comparison of age at admission

Appendix 2 – data cleaning of routine data before professing by NHS Grouper software

Apart from routine data cleaning instances such as the recoding of string variables to numeric variables (where appropriate), the following describes other preparatory or pre-processing changes made to data received from hospital prior to its processing by the Grouper software.

Admitted patient care dataset:

- Missing “epiorder” variables (which describe the order of episodes in a spell for an individual) were calculated from a count of episodes for individuals based on reported admission dates.
- Missing “speldur” variables (which measure the duration of spells of care) were calculated for the first episode as the difference between episode start date and episode end date
- Missing “classpat” variables (patient classification into day cases, ordinary admissions, etc) were recoded to a value identifying an “ordinary admission”, which accounted for 99.76% of all non-missing instances of this variable
- The “epidur” variable (measuring episode duration) was calculated, following instructions in the HES Data Dictionary, as the difference in days between episode start date and episode end date. One site did not report episode start dates, and the epidur variable was calculated as the difference in days between admission date and episode end date

- Missing or ungroupable “mainspef” variables (describing the main speciality under which a consultant is contracted) or “tretspef” variables (describing the speciality in which the consultant was working during the period of care) were recoded to the “General Medicine” speciality.

Critical care:

- Missing “ccunitfun” variables (describing the function of the critical care unit) were recoded to “Non-specific, general adult critical care”, which was the modal function recorded in non-missing cases

Out-patient:

- Missing or ungroupable “mainspef” variables (describing the main speciality under which a consultant is contracted) or “tretspef” variables (describing the speciality in which the consultant was working during the period of care) were recoded to the “General Medicine” speciality

Emergency:

- Missing or “aepatgroup” variables (describing the reason for an ED episode) were recoded to “Other than above”, i.e. not due to one of the named reasons for an ED episode such as road traffic accident or assault.

Appendix 3 Costing of elements of COPD admission and discharge care bundles

Tables A3.1 and A3.2 summarise how elements of care bundles were assigned costs.

Table A3.1: Strategy used to cost elements of a COPD admission care bundle

| **Admissions bundle** | |
| --- | --- |
| **Bundle element** | **Approach to costing** |
| 1. Ensure correct diagnosis of AECOPD with both: | |
| a. Chest x-ray result documented in notes within 4 hours | Cost as “Direct Access Plain Film” (currency code DAPF) from 2015/16 NHS Reference Costs (1).  Unit cost in 2015/16 prices: £30.00 |
| b. ECG result documented in the notes within 4 hours | Calculated as the and directly accessed diagnostic services “Electrocardiogram Monitoring or Stress Testing” (currency code EY51Z) currency code reported in 2015/16 NHS Reference Costs  Unit cost in 2015/16 prices: £40.00 |
| 2. Recognise and respond to respiratory acidosis within 3 hours of admission | |
| a. Arterial blood gas within 1hour if oxygen sats <94% on air or controlled oxygen | Cost as “Oximetry or blood gas studies” from NHS Reference Costs  Unit cost in 2015/16 prices: £55.00 |
| b. when pH<7.35 assess suitability for non-invasive ventilation (NIV) and implement within 3 hours of admission | Cost as “Oximetry or blood gas studies” from NHS Reference Costs, unless bundle element had already been recorded as provided – this avoids possible double counting of this element  AND  Cost of NIV: The cost of NIV was taken from “An Outcomes Strategy for COPD and Asthma”(2) and inflated to 2015/16 prices using the hospital and community health services (HCHS) index reported in Curtis and Burns (3).  Unit cost in 2015/16 prices: £55.00 for oximetry and £324.96 for NIV |

| **Admissions bundle** *(continued)* | |
| --- | --- |
| **Bundle element** | **Approach to costing** |
| 3. Recognition of hypoxia and correct oxygen prescription within 30 minutes of admission with target range of 88-92% | “Oximetry or blood gas studies”, from NHS Reference Costs, unless bundle element had already been recorded as provided – this avoids possible double counting of this element.  AND  Oxygen costs taken from estimates of cost per day from Hertel et al (4), inflated to 2015/16 prices using the hospital and community health services (HCHS) index reported in Curtis and Burns (3).  Unit cost in 2015/16 prices: £55.00 for oximetry and £16.25 for oxygen |
| 4. Correct prescription of medication for AECOPD at admission | |
| a. Steroids prescribed and administered within 4 hours of admission when necessary  b. Antibiotics prescribed and administered within 4 hours of admission when necessary  c. Nebulisers prescribed and administered within 4 hours of admission when necessary | Cost as prednisolone 5mg tablets x 42 from NHS Drug Tariff database (5). Unit cost in 2015/16 prices: £1.86  It was assumed that 10% of admissions requiring antibiotics would receive them intravenously as 1.2g of co-amoxiclav and 500mg clarithromycin. All other patients would receive amoxicillin 500mg capsules. Costs from NHS Drug Tariff database and British National Formulary. Weighted unit cost 2015/16 prices: £4.92  Nebulised therapy costed as ipratropium 500mcg and salbutamol 2.5mg. Costs from NHS Drug Tariff database. Unit cost in 2015/16 prices: £5.31 |
|  |  |
|  |  |
| 5. Review by respiratory specialist (specialist nurse, doctor or physiotherapist) within 24 hours | Unweighted average of cost of 15 minutes of time from: hospital Band 6 specialist nurse, consultant doctor or Band 6 hospital physiotherapist from Curtis and Burns (3)  Unit cost in 2015/16 prices: £45.25 |
|  |  |

Table A3.2: Sources used for costing elements of a COPD discharge care bundle

| **Discharge bundle** | |
| --- | --- |
| **Bundle element** | **Approach to costing** |
| 1. Assess prior to discharge: | |
| a. Respiratory medicines | As for 4(a) and 4(b) in admissions bundles, but excluding costs of intravenous drugs.  Unit cost in 2015/16 prices: £2.98 |
|  |  |
| b. Inhaler technique | Cost as 15 minutes of time from hospital-based Band 6 specialist nurse, from Curtis and Burns (3)  Unit cost in 2015/16 prices: £8.75 |
|  |  |
| 2. All patients should receive | |
| a. Written pack for how to manage further AECOPD | Assume zero cost |
| b. Discharge pack of emergency drugs | Assume pack comprises steroid tablets (Prednisolone 5mg tablets x 42) and antibiotic capsules (Amoxicillin 500mg capsules x 15).  Cost using NHS Drug Tariff database (5)  Unit cost in 2015/16 prices: £2.98 |
|  |  |
| 3. Assess smoking status and assess willingness to quit and – for those patients indicating a wish for further assistance – refer to a stop smoking programme | Cost as “Alveolar Carbon Monoxide Measurement or Smoking Cessation Support” for assessment, and cost referral to a nurse-led smoking cessation programme using “A ten-minute opportunistic brief advice session” reported in Curtis and Burns (3)  Unit cost in 2015/16 prices: £22.00 for assessment, £22.00 for referral |
|  |  |
|  |  |

| **Discharge bundle** *(continued)* | |
| --- | --- |
| **Bundle element** | **Approach to costing** |
| 4. Assess for suitability for pulmonary rehabilitation | Cost as 10 minutes of time from hospital-based Band 6 specialist nurse in Curtis and Burns (3)  Unit cost in 2015/16 prices: £5.83 |
|  |  |
| 5. Organise community follow up within two weeks of discharge from hospital^*^ | Cost as 45 minutes of time (based on Jordan et al (6)) from Band 6 community nurse using Curtis and Burns (3)  Unit cost in 2015/16 prices: £33.00 |
|  |  |

Appendix 4

Table A4.1: Results from available case SUR regressions

| **Models estimated** | **Seemingly unrelated regression, unadjusted model**  N=12,532 | | | **Seemingly unrelated regression, adjusted for month in year and mixed effect for trust cluster**  N=12,532 | | | **Seemingly unrelated regression, adjusted for month in year and mixed effect for trust cluster and all baseline covariates**  N=12,532 | | |
| --- | --- | --- | --- | --- | --- | --- | --- | --- | --- |
|  | **Comparator mean** | **Implementation mean** | **Interaction (95% CI)** | **Comparator mean** | **Implementation mean** | **Interaction (95% CI)** | **Comparator mean** | **Implementation mean** | **Interaction (95% CI) ^1^** |
| **Costs and 90-day survival** | | | | | | | | | |
| NHS costs in “pre” period | £5,989 | £6,707 | -£765  (-£1,359 to -£172) | £5,907 | £6,188 | -£666  (-£1,257 to -£75) | £5,733 | £6,137 | -£761  (-1,368 to -154) |
| NHS costs in “post” period | £4,760 | £4,712 |  | £4,625 | £4,240 |  | £4,475 | £4,119 |  |
|  |  |  |  |  |  |  |  |  |  |
| 90-day survival in “pre” period | 0.90 | 0.92 | 0.00  (-0.02 to 0.02) | 0.90 | 0.92 | -0.00  (-0.02 to 0.01) | 0.90 | 0.92 | -0.01  (-0.03 to 0.01) |
| 90-day survival in “post” period | 0.91 | 0.93 |  | 0.91 | 0.93 |  | 0.92 | 0.93 |  |
|  |  |  |  |  |  |  |  |  |  |
| **Cost-effectiveness statistics^1^** | | | | | | | | | |
| NMB at λ = £5,000 (95% CI) | |  | 772 (173 to 1,371) |  |  | 642 (46 to 1,239) |  |  | 731 (118 to 1,344) |
| Probability cost-effective at λ = £5,000 | | | 0.99 |  |  | 0.98 |  |  | 0.99 |
| NMB at λ = £10,000 (95% CI) | | | 778 (158 to 1,399) |  |  | 618 (0 to 1,236) |  |  | 701 (67 to 1,336) |
| Probability cost-effective at λ = £10,000 | | | 0.99 |  |  | 0.98 |  |  | 0.98 |
| NMB at λ = £20,000 (95% CI) | | | 791 (86 to 1,495) |  |  | 570 (-132 to 1,271) |  |  | 641 (-78 to 1,360) |
| Probability cost-effective at λ = £20,000 | | | 0.99 |  |  | 0.94 |  |  | 0.96 |
| NMB at λ = £30,000 (95% CI) | | | 803 (-24 to 1,631) |  |  | 521 (-305 to 1,347) |  |  | 582 (-262 to 1,426) |
| Probability cost-effective at λ = £30,000 | | | 0.97 |  |  | 0.89 |  |  | 0.91 |
| NMB at λ = £50,000 (95% CI) | | | 828 (-310 to 1,966) |  |  | 424 (-715 to 1,564) |  |  | 462 (-698 to 1,622) |
| Probability cost-effective at λ = £50,000 | | | 0.92 |  |  | 0.77 |  |  | 0.78 |

Notes: (1) Threshold values represent cost per death avoided at 90 days.

λ = cost-effectiveness threshold value. CI = Confidence interval; CE = Cost-effectiveness; NMB = Net monetary benefit

Figure A4.1: CEACs for available case SUR models

Estimated net benefit declines with increases in the threshold. This effect can be seen using the CEACs, which plot the probability of the care bundles being cost-effective for various levels of the cost-effectiveness threshold.

The imputed cost-effective results for SUR on unadjusted cases are presented below.

Table A4.2: Cost-effectiveness results – imputed cases using unadjusted

| **Model estimated** | | **Seemingly unrelated regression, unadjusted model**  N=12,532 imputed cases | |
| --- | --- | --- | --- |
|  | **Comparator mean** | **Implementation mean** | **Interaction (95% CI)** |
| **Costs and 90-day survival** |  |  |  |
| NHS costs in “pre” period | £7,398 | £6,070 |  |
| NHS costs in “post” period | £6,057 | £4,473 |  |
|  |  |  | -£256 (-£971 to £458) |
| 90-day survival in “pre” period | 0.90 | 0.92 |  |
| 90-day survival in “post” period | 0.91 | 0.93 |  |
|  |  |  | -0.01 (-0.04 to 0.02) |
| **Cost-effectiveness statistics^1^** |  |  |  |
| NMB at λ = £5,000 (95% CI) |  |  | 263 (-457 to 983) |
| Probability cost-effective at λ = £5,000 |  |  | 0.76 |
| NMB at λ = £10,000 (95% CI) |  |  | 269 (-469 to 1,008) |
| Probability cost-effective at λ = £10,000 |  |  | 0.76 |
| NMB at λ = £20,000 (95% CI) |  |  | 282 (-530 to 1,093) |
| Probability cost-effective at λ = £20,000 |  |  | 0.75 |
| NMB at λ = £30,000 (95% CI) |  |  | 294 (-627 to 1,215) |
| Probability cost-effective at λ = £30,000 |  |  | 0.73 |
| NMB at λ = £50,000 (95% CI) |  |  | 319 (-891 to 1,529) |
| Probability cost-effective at λ = £50,000 |  |  | 0.70 |

The results of the SUR models applied to imputed data suggests lower probabilities of care bundles being cost-effective than estimated using available case data.

Appendix 5 Model fit for adjusted net benefit regression on available cases

Figure A5.1 shows fitted versus predicted values for the fully adjusted available case net benefit regression model.

Figure A5.1 Fitted versus predicted values for adjusted net benefit regression on available cases


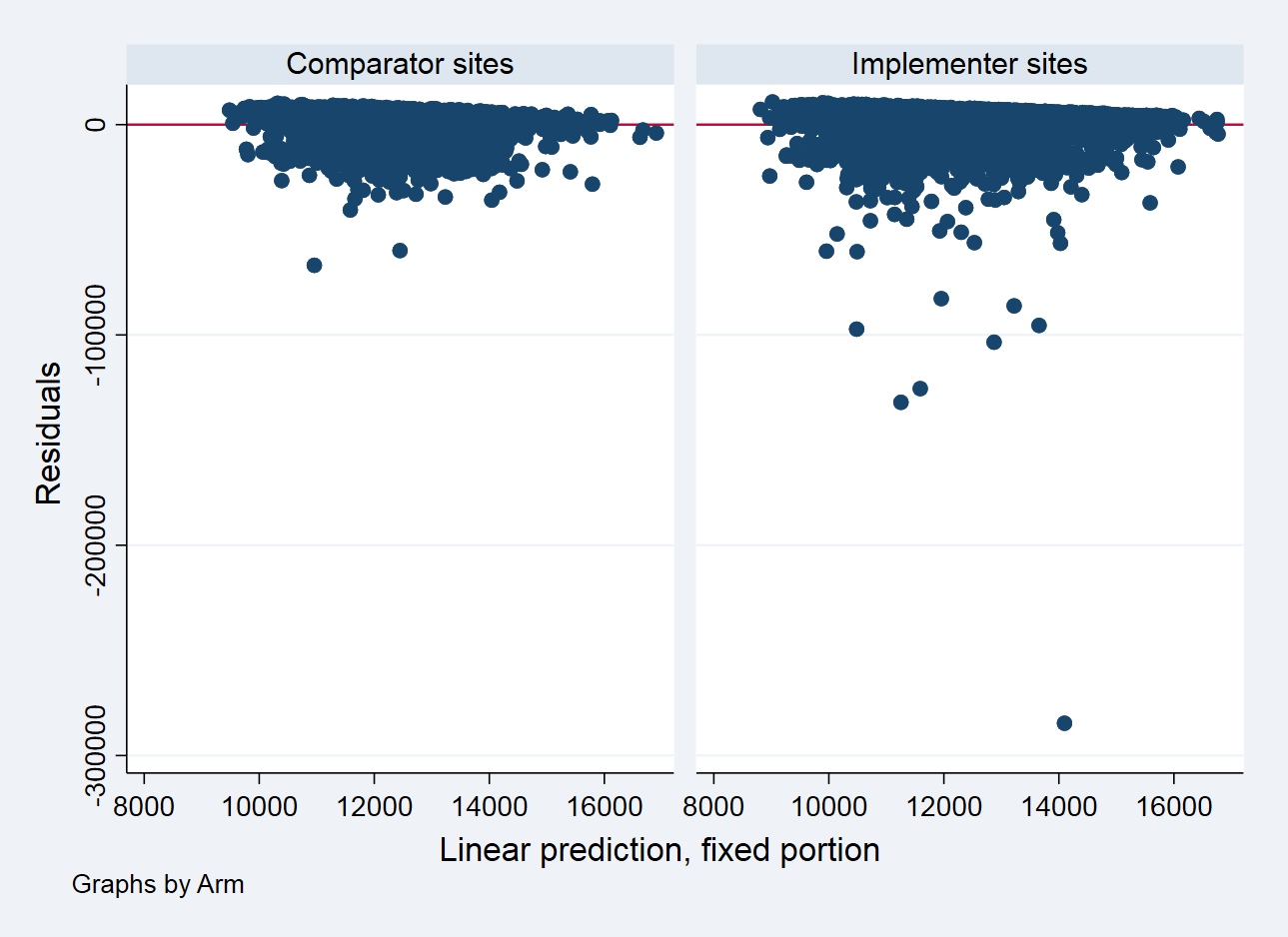


Appendix 6 Methods and results for qualitative analysis

Observation of patient interactions

The interactions between hospital staff and 19 patients were observed at five hospital sites (2 comparator, 3 implementation) for up to two hours. Of these patients, 53% (n=10) were female, and 26% (n=5) were observed following admission (rather than prior to discharge).

The principal focus of the patient observation was on clinician time administering care at admission and discharge. In the event, there was limited doctor engagement in the periods of observation reviewed at either type of site – 63% (n=12) of observations recorded no doctor contact whatsoever. Of the recorded doctor interactions (n=7), 4 occurred as part of ward rounds (all at the same site) and the remaining engagements were contacts with junior doctors that lasted on average no more than two minutes.

Other interactions observed typically included routine contacts with healthcare assistants, ward and respiratory nurses, and occasional interactions with other specialised staff, such as physiotherapists and pharmacists. The duration of these interactions was short (often less than a minute) except for occasional instances of longer engagements to review medication or to undertake spirometry. All recorded doctor interactions occurred at 2 of the 3 comparator sites, but the comparison with practices at implementation sites is complicated by the coincidental overlap of ward rounds with the period of research observation.

Post-discharge resource use

Nine patients were interviewed post-discharge – three from comparator sites and six from implementation sites. All comparator site patients reported some contact with healthcare professionals. Two of the three comparator site patients reported contact with GPs. Only one comparator-group patient reported participation in a pulmonary rehabilitation programme. Two patients reported contact from hospital following the index discharge. All patients had contact with nurses, either by telephone following the index discharge, or in person at home from community nurses.

Two of the six implementation site patients reported participation in pulmonary rehabilitation, with a third planning to attend shortly after the telephone interview. Four of the six implementation site patients reported contacts with nurses, four with GPs and one with a physiotherapist.

Implications of patient observation and interviews cost-effectiveness analysis

Overall, there is no evidence of gross differences in doctor or other clinician engagement between implementation and comparator sites, or in relation to intensity of resource use post-discharge. We had planned (7) to assign costs to these interactions based on the unit cost per hour of the professionals involved with patient interaction. However, in a number of cases these costs would have amounted to £1 or less per interaction, and we concluded that the exercise was unlikely to be informative of resource differences between site types.

Appendix 7 Completed CHEERS Checklist

The CHEERS checklist lists all items which should be included when reporting economic evaluations of health interventions.

| **Section / item** | **Item** | **Recommendation** | **Reported on page / line** |  |
| --- | --- | --- | --- | --- |
| **Title and abstract** | | | | |
| Title | 1 | Identify the study as an economic evaluation or use more specific terms such as “cost-effectiveness analysis”, and describe the interventions compared. | P1 |  |
| Abstract | 2 | Provide a structured summary of objectives, perspective, setting, methods (including study design and inputs), results (including base case and uncertainty analyses), and conclusions. | P 2 |  |
| **Introduction** | | | | |
| Background and objectives | 3 | Provide an explicit statement of the broader context for the study. | P3-4 |  |
|  |  | Present the study question and its relevance for health policy or practice decisions. | P3-4 |  |
| **Methods** | | | | |
| Target population and subgroups | 4 | Describe characteristics of the base case population and subgroups analysed, including why they were chosen. | P4-5 |  |
| Setting and location | 5 | State relevant aspects of the system(s) in which the decision(s) need(s) to be made. | P4-5 |  |
| Study perspective | 6 | Describe the perspective of the study and relate this to the costs being evaluated. | P5 |  |
| Comparators | 7 | Describe the interventions or strategies being compared and state why they were chosen. | P3/4 |  |
| Time horizon | 8 | State the time horizon(s) over which costs and consequences are being evaluated and say why appropriate. | P5 |  |
| Discount rate | 9 | Report the choice of discount rate(s) used for costs and outcomes and say why appropriate. | P5 |  |
| Choice of health outcomes | 10 | Describe what outcomes were used as the measure(s) of benefit in the evaluation and their relevance for the type of analysis performed. | P7 |  |
| Measurement of effectiveness | 11a | *Single study-based estimates:*Describe fully the design features of the single effectiveness study and why the single study was a sufficient source of clinical effectiveness data. | P4-9 |  |
|  | 11b | *Synthesis-based estimates*: Describe fully the methods used for identification of included studies and synthesis of clinical effectiveness data. | N/A |  |
| Measurement and valuation of preference based outcomes | 12 | If applicable, describe the population and methods used to elicit preferences for outcomes. | N/A |  |
| Estimating resources  and costs | 13a | *Single study-based economic evaluation:* Describe approaches used to estimate resource use associated with the alternative interventions. Describe primary or secondary research methods for valuing each resource item in terms of its unit cost. Describe any adjustments made to approximate to opportunity costs. |  |  |
|  | 13b | *Model-based economic evaluation:*  Describe approaches and data sources used to estimate resource use associated with model health states. Describe primary or secondary research methods for valuing each resource item in terms of its unit cost. Describe any adjustments made to approximate to opportunity costs. | N/A |  |
| Currency, price, date  and conversion | 14 | Report the dates of the estimated resource quantities and unit costs. Describe methods for adjusting estimated unit costs to the year of reported costs if necessary. Describe methods for converting costs into a common currency base and the exchange rate. | P5-7 |  |
| Choice of model | 15 | Describe and give reasons for the specific type of decision-analytical model used. Providing a figure to show model structure is strongly recommended. | N/A |  |
| Assumptions | 16 | Describe all structural or other assumptions underpinning the decision-analytical model. | N/A |  |
| Analytical methods | 17 | Describe all analytical methods supporting the evaluation. This could include methods for dealing with skewed, missing, or censored data; extrapolation methods; methods for pooling data; approaches to validate or make adjustments (such as half cycle corrections) to a model; and methods for handling population heterogeneity and uncertainty. | P9-12 |  |
| **Results** | | | | |
| Study parameters | 18 | Report the values, ranges, references, and, if used, probability distributions for all parameters. Report reasons or sources for distributions used to represent uncertainty where appropriate. Providing a table to show the input values is strongly recommended. | P13-15 |  |
| Incremental costs  and outcomes | 19 | For each intervention, report mean values for the main categories of estimated costs and outcomes of interest, as well as mean differences between the comparator groups. If applicable, report incremental cost-effectiveness ratios. | P13-15 |  |
| Characterising uncertainty | 20a | *Single study-based economic evaluation:* Describe the effects of sampling uncertainty for the estimated incremental cost and incremental effectiveness parameters, together with the impact of methodological assumptions (such as discount rate, study perspective). | P17 |  |
|  | 20b | *Model-based economic evaluation:*Describe the effects on the results of uncertainty for all input parameters, and uncertainty related to the structure of the model and assumptions. | N/A |  |

| Characterising heterogeneity | 21 | If applicable, report differences in costs, outcomes, or cost-effectiveness that can be explained by variations between subgroups of patients with different baseline characteristics or other observed variability in effects that are not reducible by more information. | N/A |  |
| --- | --- | --- | --- | --- |
| **Discussion** | | | | |
| Study findings, limitations, generalisability, and current knowledge | 22 | Summarise key study findings and describe how they support the conclusions reached. Discuss limitations and the generalisability of the findings and how the findings fit with current knowledge. | P23-24 |  |
| **Other** | | | | |
| Source of funding | 23 | Describe how the study was funded and the role of the funder in the identification, design, conduct, and reporting of the analysis. Describe other non-monetary sources of support. | P25/26 |  |
| Conflicts of interest | 24 | Describe any potential for conflict of interest of study contributors in accordance with journal policy. In the absence of a journal policy, we recommend authors comply with International Committee of Medical Journal Editors recommendations. | COI forms attached |  |

References

1. Department of Health. Reference Costs 2015/16. 2016.

2. Department of Health. An Outcomes Strategy for COPD and Asthma: NHS Companion Document. London: NHS, **2012**.

3. Curtis L, Burns A. Unit Costs of Health and Social Care 2016. Canterbury: University of Kent, 2016.

4. Hertel N, Kotchie RW, Samyshkin Y, et al. Cost-effectiveness of available treatment options for patients suffering from severe COPD in the UK: a fully incremental analysis. International Journal of Chronic Obstructive Pulmonary Disease. 2012; 7: 183-99.

5. NHS. Drug tariff. Various years,.

6. Jordan RE, Majothi S, Heneghan NR, et al. Supported self-management for patients with moderate to severe chronic obstructive pulmonary disease (COPD): an evidence synthesis and economic analysis. Health Technol Assess. 2015; 19.

7. Chalder MJE, Wright CL, Morton KJP, et al. Study protocol for an evaluation of the effectiveness of ‘care bundles’ as a means of improving hospital care and reducing hospital readmission for patients with chronic obstructive pulmonary disease (COPD). BMC Pulmonary Medicine. 2016; 16: 35.
